# A new prognostic model for recurrent pregnancy loss: assessment of thyroid and thromboelastograph parameters

**DOI:** 10.3389/fendo.2024.1415786

**Published:** 2024-05-30

**Authors:** Fangxiang Mu, Huyan Huo, Chen Wang, Ning Hu, Fang Wang

**Affiliations:** Department of Reproductive Medicine, Lanzhou University Second Hospital, Lanzhou, China

**Keywords:** recurrent pregnancy loss, thyroid function, thromboelastograph, nomogram, prediction model

## Abstract

**Objective:**

This study aimed to identify predictors associated with thyroid function and thromboelastograph (TEG) examination parameters and establish a nomogram for predicting the risk of subsequent pregnancy loss in recurrent pregnancy loss (RPL).

**Methods:**

In this retrospective study, we analyzed the medical records of 575 RPL patients treated at Lanzhou University Second Hospital, China, between September 2020 and December 2022, as a training cohort. We also included 272 RPL patients from Ruian People’s Hospital between January 2020 and July 2022 as external validation cohort. Predictors included pre-pregnancy thyroid function and TEG examination parameters. The study outcome was pregnancy loss before 24 weeks of gestation. Variable selection was performed using least absolute shrinkage and selection operator regression and stepwise regression analyses, and the prediction model was developed using multivariable logistic regression. The study evaluated the model’s performance using the area under the curve (AUC), calibration curve, and decision curve analysis. Additionally, dynamic and static nomograms were constructed to provide a visual representation of the models.

**Results:**

The predictors used to develop the model were body mass index, previous pregnancy losses, triiodothyronine, free thyroxine, thyroid stimulating hormone, lysis at 30 minutes, and estimated percent lysis which were determined by the multivariable logistic regression with the minimum Akaike information criterion of 605.1. The model demonstrated good discrimination with an AUC of 0.767 (95%CI 0.725-0.808), and the Hosmer-Lemeshow test indicated good fitness of the predicting variables with a *P* value of 0.491. Identically, external validation confirmed that the model exhibited good performance with an AUC of 0.738. Moreover, the clinical decision curve showed a positive net benefit in the prediction model. Meanwhile, the web version we created was easy to use. The risk stratification indicated that high-risk patients with a risk score >147.9 had a higher chance of pregnancy loss (OR=6.05, 95%CI 4.09-8.97).

**Conclusions:**

This nomogram well-predicted the risk of future pregnancy loss in RPL and can be used by clinicians to identify high-risk patients and provide a reference for pregnancy management of RPL.

## 1 Introduction

Recurrent pregnancy loss (RPL) is defined as two or more pregnancy losses before 24^th^ weeks of gestation, whether consecutive or non-consecutive, excluding ectopic and molar pregnancies, and implantation failure, according to the European Society of Human Reproduction and Embryology (ESHRE) guideline (1). It affects approximately 1%~5% women of childbearing age (2). Research indicates that the risk of RPL increases with the number of previous pregnancy losses. For individuals who have experienced three or more pregnancy losses, the chances of experiencing another pregnancy loss after a second pregnancy range from 40% to 80% (3). The etiology of RPL is complex and remains unexplained in nearly 50% of cases. Various factors have been investigated as potential causes of RPL, including immune factors, endocrine disorders, uterus anatomical abnormalities, and pre-thrombotic state (PTS) (4–6). Immune factors play a significant role in RPL. They may contribute to placental abnormal development and damage by affecting endometrial angiogenesis and activating immune complexes and the complement system (7, 8). Studies have shown that anti-β2 glycoprotein I antibodies that may indirectly promote RPL by reducing the Bcl-2/Bax ratio and affecting critical cell survival pathways (9). Besides, the expression of human leukocyte antigen (HLA)-G and HLA-C has been observed to be abnormal in RPL patients, which may interfere with fetal immune tolerance and further exacerbate the occurrence of RPL (10).

Additionally, studies have demonstrated that thyroid status is associated with an elevated risk of pregnancy loss (11). Thyroid dysfunction is a common endocrine disorder that impacts 2-3% of females in their childbearing years and has been recognized as a potential contributor to RPL (12). Additionally, higher levels of thyroid-stimulating hormone (TSH) during pregnancy were found to increase the risk of pregnancy loss by 60% (13). A comparison of thyroid function between non-pregnant RPL women and age-matched controls demonstrated a higher incidence of hypothyroidism among RPL women (4.29%) as compared to controls (0.61%) (14). Screening for pre-pregnancy thyroid dysfunction in RPL patients has been proposed but has not been supported by adequate research (1, 15).

Thrombelastograph (TEG) is a test that evaluates the dynamic process of coagulation and fibrinolysis as a whole, and it can monitor the entire coagulation process dynamically using only a small amount of the patient’s whole blood. TEG has been widely used and has been proven effective in monitoring coagulation and fibrinolysis during extracorporeal circulation, diffuse endovascular coagulation, and renal transplantation (16, 17). PTS is the tendency of persistent hypercoagulable clots to cause thrombosis, a pathological process causing an imbalance in the hemostatic, coagulation, and anticoagulation processes, which is closely related to the development of RPL (18). Studies have shown that 50% to 65% of pregnant women with RPL have a hypercoagulable state of the blood, which increases the resistance to blood flow in the uterine and placental arteries, thrombosis in the microvessels, and insufficient perfusion of intraplacental blood flow, leading to pregnancy loss due to cessation of development of the embryo or fetus due to ischemia (19, 20). However, TEG has been little studied in RPL (21, 22), especially the application of its parameters in assessing the outcome of subsequent pregnancies (23). Rai et al. found that the future pregnancy outcome of RPL could be predicted by the pre-pregnancy maximum amplitude (MA) value with an area under the curve (AUC) of 0.7 (95%CI 0.59-0.82) (23), suggesting the potential value of pre-pregnancy TEG parameters in predicting subsequent pregnancy outcomes in RPL patients warrants further investigation.

Previous predictive models for RPL’s pregnancy outcomes have concentrated on conventional risk factors like maternal age, reproductive history, and previous pregnancy losses. However, they have not adequately considered other factors that may impact pregnancy outcomes, like thyroid peroxidase antibodies (TPOAb), TSH, and TEG parameters. This limits the need for optimization of management strategies for future pregnancy in RPL patients. In addition, some predictive models developed based on immunologic factors may be costly for the patients, whereas thyroid function tests and TEG tests are clinically valid, reliable, and cost-effective predictors. Also, it is important to note that some studies have limitations, such as a lack of external validation (24, 25). These factors limited the generalizability of the models and their applicability in clinical practice. Therefore, we propose to develop a prediction model that includes parameters from pre-pregnancy thyroid function and TEG tests to evaluate the risk of subsequent pregnancies in RPL patients. This will facilitate timely and appropriate interventions to improve the outcome of RPL pregnancies.

This study aimed to identify variables linked to pre-pregnancy thyroid function and TEG examinations and further developed a prediction model as a scientific and convenient instrument to evaluate the risk of subsequent pregnancy loss in RPL patients. This work can be a useful reference for the management of RPL patients’ future pregnancies.

## 2 Methods

### 2.1 Participants

**Figure 1:**
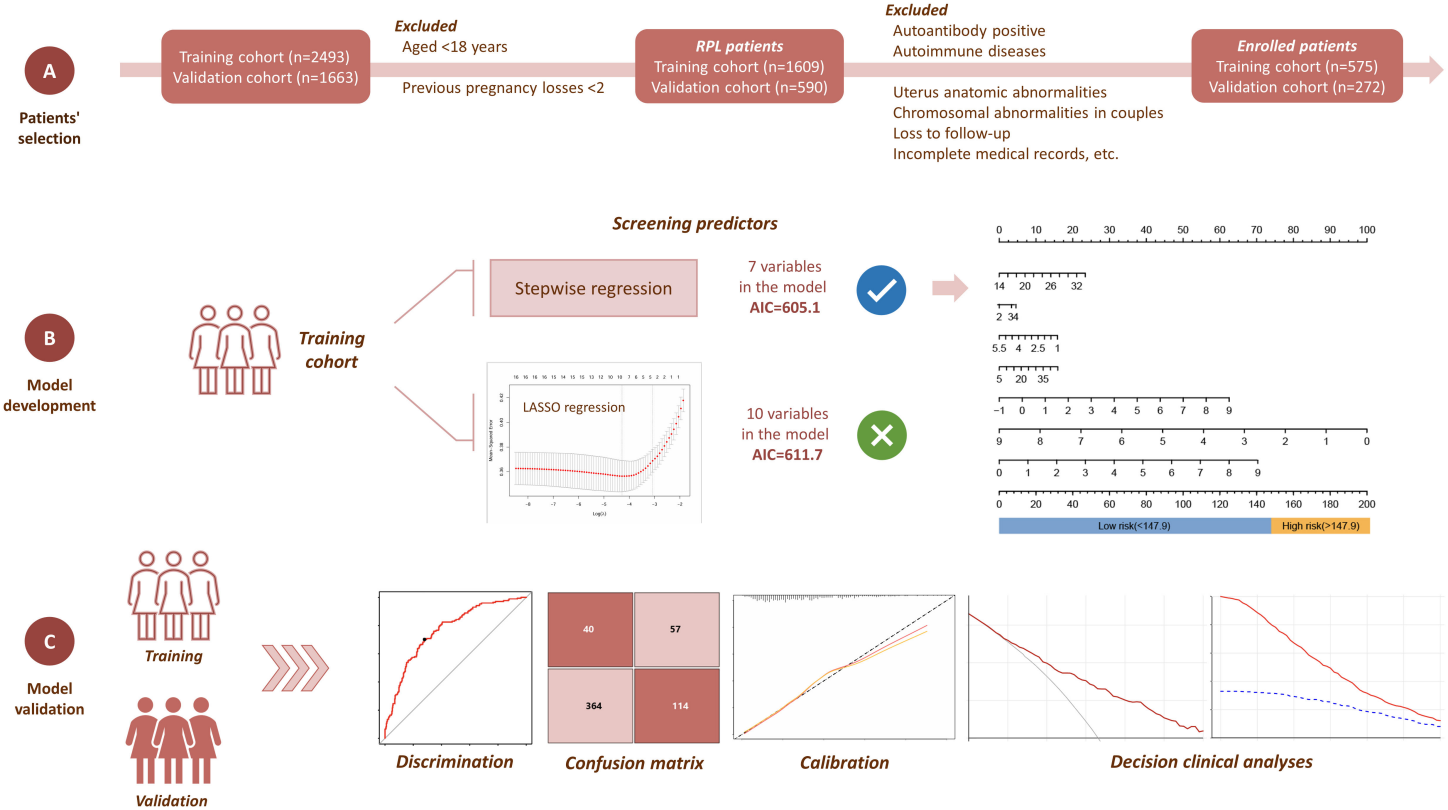
Overview of the research workflow.

To conduct this research, individuals diagnosed with RPL were recruited from the Department of Reproductive Medicine, Lanzhou University Second Hospital, between September 2020 and December 2022. Additionally, RPL patients from Ruian People’s Hospital between January 2020 and July 2022 were integrated into the external validation cohort. The diagnostic work-up was mostly recommended by the most recent ESHRE guidelines (1). **Figure 1** presents the overall flowchart of the study. All procedures of the study were approved by the Ethics Committee of the Lanzhou University Second Hospital (Approval No. 2019A-231).

The study included participants who met the following criteria: (1) Age of ≥18 years; (2) Experienced a history of at least two pregnancy losses before 24^th^ weeks of gestation. We also applied the following exclusion criteria for both the training cohort and validation cohort: (1) Parental chromosomal abnormalities; (2) Autoimmune diseases, such as antiphospholipid syndrome, systemic lupus erythematosus, and Sjögren’s syndrome; (3) Hyperthyroidism, hypothyroidism, and subclinical hypothyroidism; (4) Polycystic ovary syndrome; (5) Anatomic abnormalities of the uterus; (6) Autoantibody test positive, such as antiphospholipid antibodies, anti-β2 glycoprotein antibodies, lupus anticoagulant, and antinuclear antibodies; (7) Infertility; (8) Adverse pregnancy outcomes such as molar pregnancy and ectopic pregnancy; (9) Lack of necessary follow-up information; (10) Absence of necessary pre-pregnancy thyroid function or TEG tests; (11) No fertility plan.

### 2.2 Definition and follow-up

The study outcome was pregnancy loss, determined through a comprehensive review of both medical records and telephone follow-up after 11 months of pregnancy diagnosis. Pregnancy loss refers to the spontaneous abortion that occurs before 24 weeks of gestation.

### 2.3 Candidate predictive variables

The following candidate variables were involved in this study: (1) Demographic characteristics, including age, history of previous pregnancy losses, and body mass index (BMI); (2) Parameters for testing thyroid function, including TSH, triiodothyronine (T3), free T3 (FT3), thyroxine (T4), free T4 (FT4), thyroglobulin (TG), antithyroglobulin antibody (TGAb), and TPOAb; (3) Parameters from TEG examination, which included reaction time (R), kinetic time (K), alpha angle, MA, lysis at 30 minutes (LY30), estimated percent lysis (EPL), and coagulation index. In addition to the number of previous pregnancy losses, TGAb and TPOAb as categorical variables, and the remaining were continuous variables.

Blood samples were collected at the time of patient visits by professionals. The cobas e 411 analyzer (Roche Diagnostics International Ltd., USA) was used to perform the thyroid function test, while the TEG examination was tested using SIKMA thromboelastometer (Simes-Sikma Biotechnology Co Ltd., China).

### 2.4 Statistical analysis

To avoid degradation of model performance, we used multiple interpolations to fill in missing values. The χ^2^ test or Fisher’s exact test was employed to analyze categorical variables, and *t* test or Wilcoxon rank sum test was used to analyze continuous variables. The results were reported as frequency with percentages or mean ± standard deviation.

The study employed stepwise regression and least absolute shrinkage and selection operator (LASSO) regression to screen the predictors. Stepwise regression adopted “backward” method to screen the variables, with Akaike information criterion (AIC) as stopping rule, while LASSO regression adopted lambda.min as the criterion for variable selection. The selected variables by the two methods were then used to establish a predictive model, respectively. To avoid underfitting or overfitting, the model was considered as final model for this study based on the minimum AIC value.

Utilizing the aforementioned screening criteria, a nomogram was constructed to evaluate the likelihood of subsequent pregnancy loss in RPL. The total score corresponds to a probability of subsequent pregnancy loss. Besides, an online version of the nomogram was developed for user convenience. After assessing the risk scores that corresponded to the maximum Youden index, patients were categorized into low and high-risk groups. The difference in pregnancy loss rates between the groups was assessed using the χ^2^ test. The area under the receiver operating characteristic (ROC) curve (AUC) was employed to assess the model’s discrimination. Calibration curve and the *P* value from the Hosmer-Lemeshow test statistic were used to quantitatively assess how well the model’s predictions match actual values, indicating the model’s calibration accuracy. In addition, a decision curve analysis (DCA) has been conducted to evaluate the practical usefulness of the predictive model. This was done by computing the net benefit for various risk thresholds, and the results were visually displayed using a clinical decision curve and clinical impact curve. These curves provide an intuitive reflection of the net benefit to patients at different risk thresholds and help make informed clinical decisions by weighing the benefits of true positive and false positive.

The statistical analyses were performed utilizing SPSS software version 25.0 (IBM Corp., Armonk, NY, USA; https://www.ibm.com/spss), R studio (2022.02.1 + 461) (https://www.rstudio.com), and R version 4.1.3 (R Foundation for Statistical Computing, Vienna, Austria; http://www.R-project.org). A significance level of *P <*0.05 was considered statistically significant.

## 3 Results

A total of 575 eligible RPL cases were ultimately included in the training cohort, and 272 RPL patients were enrolled in the validation cohort. Details are shown in **Supplementary Figures S1**-**2**. There were notable differences observed in the majority of thyroid function and TEG examination parameters, in addition to maternal age, BMI, T4, TSH, TGAb, TPOAb, and R values (**Supplementary Table S1**).

### 3.1 Baseline characteristics

In **Table 1**, the basic demographic information and examination results of the enrolled patients were displayed. Out of a total of 575 patients with RPL, 404 (70%) achieved live birth, while 171 (30%) experienced pregnancy loss. There was a statistically significant difference in maternal age, previous pregnancy losses, BMI, TSH levels, LY30 values, and EPL values. The pregnancy loss group had higher maternal age (31.50 ± 4.41 vs. 30.05 ± 4.04), BMI (23.18 ± 3.05 vs. 21.97 ± 2.90) and TSH levels (2.55 ± 0.96 vs. 1.83 ± 0.90), whereas the LY30 (0.20 ± 0.51 vs. 0.49 ± 1.09) and EPL values (0.25 ± 0.59 vs. 0.51 ± 1.14) were lower (all *P <*0.05).

**Table 1:** Comparison of baseline and clinical characteristics between live birth and pregnancy loss groups in the training cohort.

Variables	Live birth (n=404)	Pregnancy loss (n=171)	*P* value
Maternal age, years	30..5±4.04	31.50±4.41	0.003
BMI, kg/m^2^	21.97±2.90	23.18±3.05	<0.001
Previous pregnancy losses			
2	312 (77.2)	115 (67.3)	0.037
3	60 (14.9)	34 (19.9)	
≥4	32 (7.9)	22 (12.9)	
T3, nmol/L	1.82±0.33	1.79±0.41	0.239
T4, nmol/L	112.00±24.45	111.69±22.11	0.888
FT3, pmol/L	5.23±0.70	5.15±0.54	0.196
FT4, pmol/L	16.31±2.75	16.33±2.32	0.921
TSH, μIU/mL	1.83±0.90	2.55±0.96	<0.001
TG, ng/mL	11.80±15.30	13.55±21.55	0.272
TGAb (+)	43 (10.6)	21 (12.3)	0.568
TPOAb (+)	42 (10.4)	24 (14.0)	0.211
R, min	5.89±2.85	5.70±1.50	0.409
K, min	3.06±8.17±	2.47±5.55	0.392
Angle, deg	63.72±11.50	64.26±9.29	0.587
MA, mm	64.39±5.80	64.95±4.64	0.260
LY30	0.49±1.09	0.20±0.51	<0.001
EPL, %	0.51±1.14	0.25±0.59	<0.001
CI, %	0.62±1.9	0.64±1.88	0.918

Data are presented as mean ± standard deviation or frequencies with percentages. BMI, body mass index; T3, triiodothyronine; T4, thyroxine; FT3 free triiodothyronine; FT4, free thyroxine; TSH, thyroid stimulating hormone, TG, thyroglobulin; TGAb, antithyroglobulin antibody; TPOAb, thyroid peroxidase antibody; R, reaction time; K, kinetic time; MA, maximum amplitude; LY30, lysis at 30 minutes; EPL, estimated percent lysis; CI, coagulation index.

### 3.2 Predictor screening

The stepwise regression analysis resulted in a model with the smallest AIC of 605.1, compared to 611.7 for the LASSO regression. Thus, we chose the variables screened by stepwise regression to establish the final prediction model. **Table 2** summarizes the variable subsets screened by each method. Ultimately, predictors for building the predictive model consisted of seven pre-pregnancy parameters: the number of previous pregnancy losses (2, 3, or ≥4), BMI, TSH, T3, T4, LY30, and EPL.

**Table 2:** Selecting predictive variables using LASSO and stepwise regression.

Variables	LASSO regression	Stepwise regression
Maternal age	True	
BMI	True	True
Previous pregnancy losses	True	True
T3	True	True
FT4	True	True
TSH	True	True
TG	True	
R	True	
K	True	
LY30	True	True
EPL		True
AIC value	611.7	605.1
Number of predictors	10	7

BMI, body mass index; T3, triiodothyronine; FT4, free thyroxine; TSH, thyroid stimulating hormone; TG, thyroglobulin; R, reaction time; K, kinetic time; LY30, lysis at 30 minutes; EPL, estimated percent lysis; AIC, Akaike information criterion.

### 3.3 Model development

**Figure 2:**
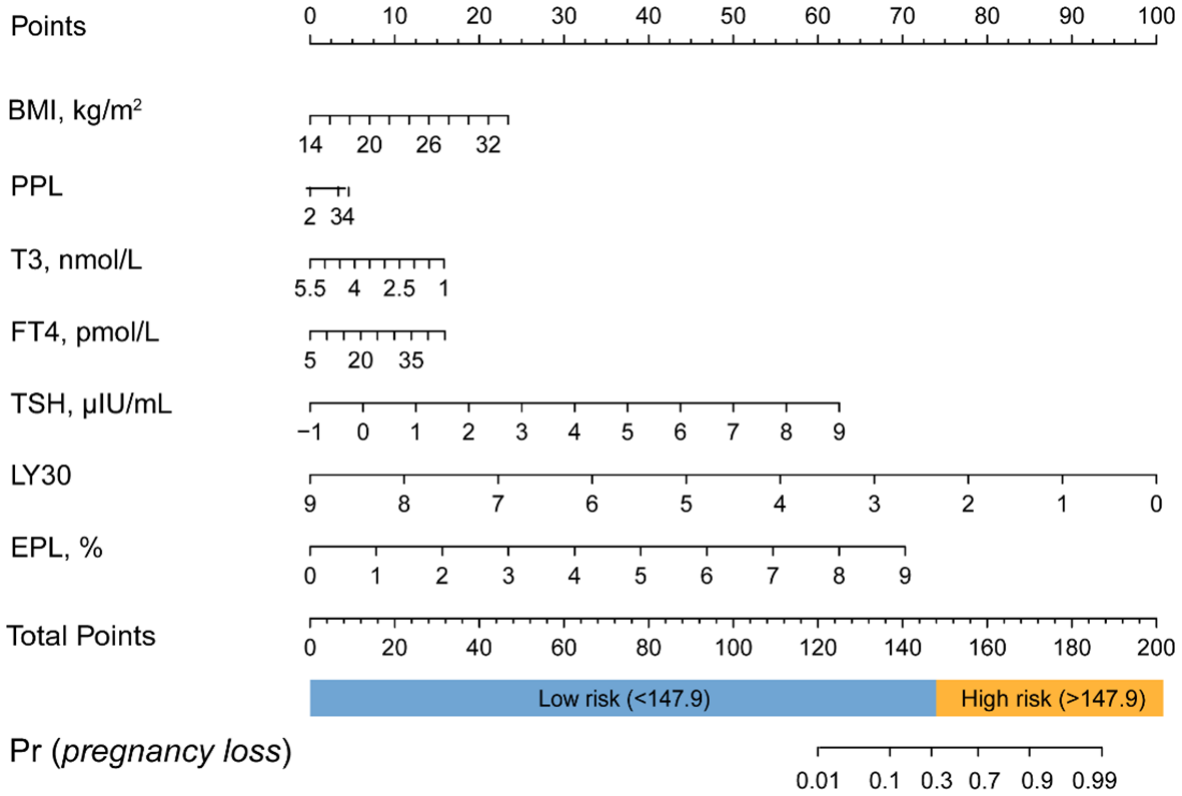
Nomogram for predicting the risk of subsequent pregnancy loss in RPL patients. PPL, previous pregnancy loss.

**Figure 3:**
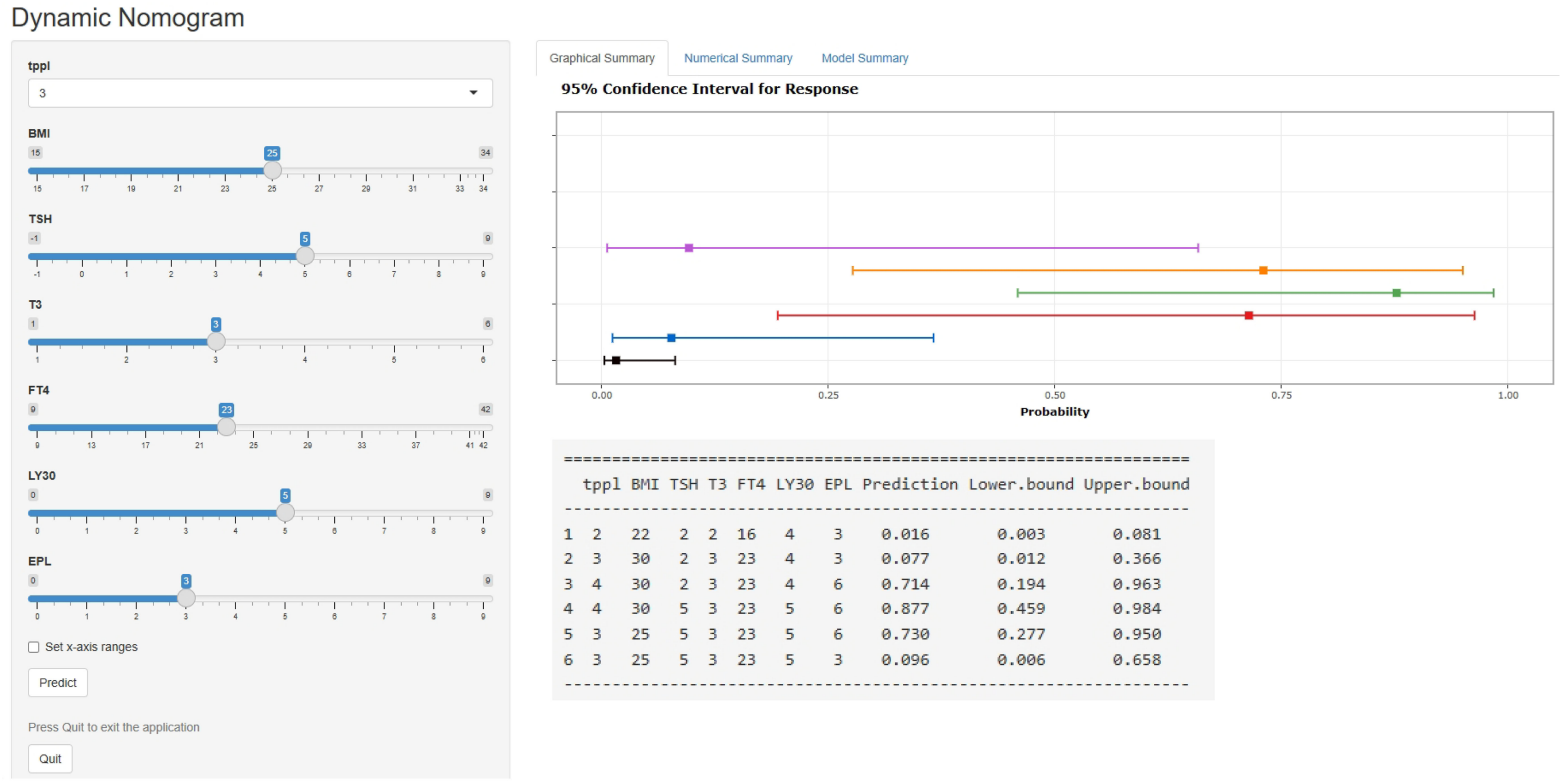
A web-based dynamic nomogram for predicting the probability of pregnancy loss in RPL patients’ subsequent pregnancies (https://zhouzhoukeyan.shinyapps.io/DynNomogram/).

**Table 3** displays the effects of the above seven predictors on the risk of subsequent pregnancy loss. Using these variables, we constructed a predictive nomogram, presented in **Figure 2**. This model quantified each variable to calculate the RPL patients’ total risk score, which corresponds to the likelihood of subsequent pregnancy loss. In addition, we calculated the total risk scores for 575 patients using the nomogram’s risk quantification and stratified them by determining the optimal cut-off score based on the ROC curve. Patients with a risk point of <147.9 (risk likelihood <30.8%) were placed in the low-risk (59.5%, 342/575) group, while the remaining patients were placed in the high-risk (40.5%, 233/575). Furthermore, individuals in the high-risk had a 6.05-fold higher risk of pregnancy loss (95%CI 4.09-8.97) compared to those in the low-risk group. An internet-based version of this nomogram is available at https://zhouzhoukeyan.shinyapps.io/DynNomogram/. It automatically calculates the pregnancy loss risk by entering the patient’s information (**Figure 3**).

**Table 3:** Multivariate logistic regression for the association between final predictors and subsequent pregnancy loss in RPL.

Variables	OR (95%CI)	*P* value
BMI	1.175 (1.110-1.258)	<0.001
Previous pregnancy losses		
Ref	–	
=3	1.583 (0.934-2.658)	0.084
≥4	1.873 (0.946-3.668)	0.068
T3	0.616 (0.333-1.086)	0.106
FT4	1.056 (0.981-1.139)	0.143
TSH	2.369 (1.891-3.002)	<0.001
LY30	0.216 (0.069-0.610)	0.046
EPL	2.938 (1.136-8.051)	0.025

Data are presented as odds ratio (OR) and 95% confidence interval (CI). BMI, body mass index; T3, triiodothyronine; FT4, free thyroxine; TSH, thyroid stimulating hormone; LY30, lysis at 30 minutes; EPL, estimated percent lysis.

### 3.4 Model validation

**Figure 4:**
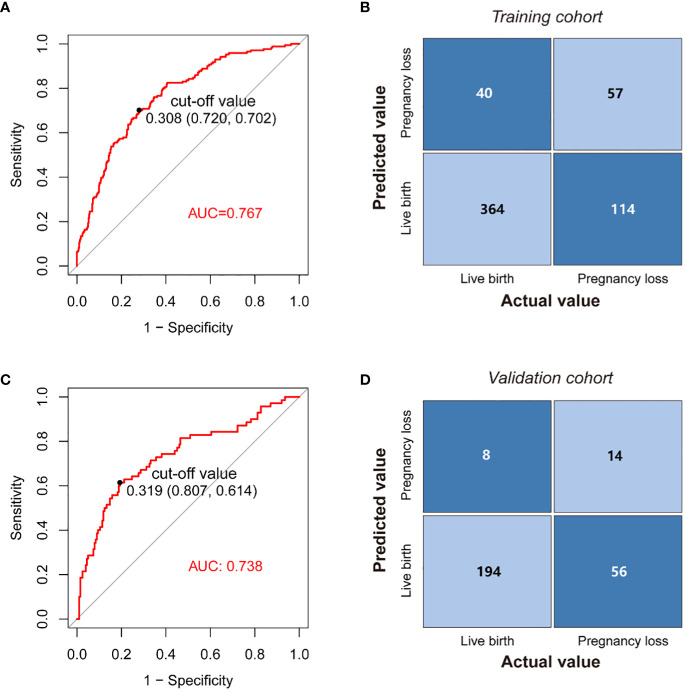
Application of the nomogram. **(A)** ROC curves of the model in the training cohort. **(B)** Confusion matrix for assessing differences between predicted risk and actual risk of subsequent pregnancy loss in the training cohort. **(C)** ROC curves of the nomogram in the validation cohort. **(D)** Confusion matrix for assessing differences between predicted risk and actual risk of subsequent pregnancy loss in the validation cohort.

**Figure 5:**
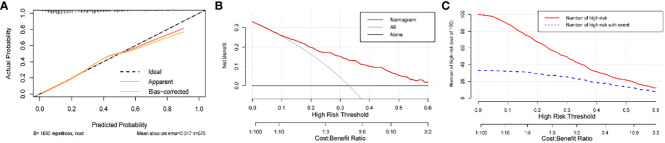
Calibration and clinical usefulness of the nomogram in the training cohort. **(A)** Calibration curve. **(B)** Clinical decision curve. For example, the model can identify around 10 cases out of 100 RPL patients that would experience pregnancy loss by setting the risk threshold at 40%, without increasing false positives. **(C)** Clinical impact curve. For instance, when predicting 100 individuals at a risk threshold of 40%, the model would determine around 31 individuals as high risk, of whom approximately 20 are true positives. The curve shows that the model can still effectively identify the individuals who will actually have pregnancy loss under the high-risk threshold setting.

The model’s AUC value in the training cohort was 0.767 (95%CI 0.725-0.808), indicating good performance in distinguishing between patients who would achieve live birth and those who would experience pregnancy loss (**Figure 4A**). As shown in the confusion matrix, the accuracy of the risk stratification was found to be 0.732 (95%CI 0.694-0.766) (**Figure 4B**). In the external validation cohort, the model’s AUC value was 0.738 (95%CI 0.665-0.810), and the accuracy of risk stratification was 0.765 (95%CI 0.711-0.811) (**Figures 4C, D**). In both the training and validation cohorts, the model’s calibration curve showed close alignment with the diagonal line, as evidenced by the *P* value of 0.491 and 0.076 in the Hosmer-Lemeshow test, respectively (**Figures 5A**, **Supplementary Figure S3A**). Finally, a DCA curve was plotted to illustrate the clinical applicability of the model (**Figure 5B**). For example, the model can identify around 10 cases out of 100 RPL patients that would experience pregnancy loss by setting the risk threshold at 40%, without increasing false positives. The relationship between the number of true positives and individuals classified as high risk at different risk thresholds is shown in **Figure 5C**. For instance, when predicting 100 individuals at a risk threshold of 40%, the model would determine around 31 individuals as high risk, of whom approximately 20 are true positives.

## 4 Discussion

In this study, we established and validated a nomogram with an online version based on pre-pregnancy thyroid function and TEG test parameters to predict the risk of subsequent pregnancy loss in RPL. The model was constructed by BMI, previous pregnancy losses, TSH, T3, T4, LY30, and EPL, and exhibited good performance, with an AUC of 0.767. Meanwhile, the model-based risk stratification constructed could well identify high-risk patients and assist clinicians in making informed decisions about RPL’s subsequent pregnancy management.

Our analysis revealed that increased BMI is a risk factor for further pregnancy loss in RPL. Several studies have demonstrated the association between obesity and pregnancy loss (26, 27). A meta-analysis showed that pregnancy loss risk was increased by 35% and 77% in the RPL population with BMI >25 kg/m^2^ and >30 kg/m^2^, respectively (28). Some factors related to obesity, such as insulin resistance and elevated levels of leptin, may contribute to pregnancy loss (29). Insulin resistance may affect pregnancy outcome in RPL by influencing the endometrial environment and embryo implantation (30). On the other hand, leptin plays a crucial role in maintaining a normal pregnancy (31). Leptin may have a positive impact on endometrial tolerance by promoting the expression of cytotrophoblast matrix metalloproteinases (32). However, as BMI increases, there may be a state of leptin resistance and a relative deficiency in women who move into the moderate to severe obesity stage, which may contribute to the risk of pregnancy loss (33). Overall, it is crucial for RPL patients to be alert to the potential risk of subsequent pregnancy loss associated with elevated BMI or obesity. Early intervention for BMI-related health problems is key to improving the outcomes of subsequent pregnancies in these patients. Furthermore, it was discovered that an increase in the number of previous pregnancy losses resulted in a higher risk of pregnancy loss in subsequent pregnancies for women with RPL. The finding was consistent with previous research (34). Current guidelines recommend making prognostic judgments based on the previous pregnancy history in the RPL patients (1). As reported, the risk of recurrence of pregnancy loss in RPL patients was more than 80%, especially in those who experienced ≥3 pregnancy losses (2).

The available evidence suggests that thyroid hormones play a crucial role in the implantation process and pregnancy maintenance. Kakita-Kobayashi et al. discovered that thyroid hormones promote the decidualization of human endometrial cells by influencing the expression of several transcription factors essential for this process (35). In the current study, the results found that pre-pregnancy higher TSH and FT4 levels, and lower T3 levels were positively linked to the subsequent pregnancy loss risk in RPL. Consistent with previous studies, an increased risk of pregnancy loss was correlated with elevated pre-pregnancy TSH levels (36). However, the exact mechanism linking thyroid hormone disorder and pregnancy loss remains unclear. TSH has been shown to enhance the expression of leukemia inhibitory factor (LIF) and its receptor in endometrial stromal cells and stimulates the expression of glucose transporter protein (GLUT1) in Ishikawa cell line, subsequently playing a role in endometrial glucose transport (37). Thus, abnormal TSH levels can affect endometrial tolerance and glucose availability, potentially leading to abnormal early embryo development, and increasing the risk of pregnancy loss. In addition, the synthesis and release of thyroid hormone into the circulation are regulated by TSH derived from pituitary. Elevated pre-pregnancy TSH levels may suggest subclinical hypothyroidism, which may affect the growth and development of the embryo in early pregnancy, thereby increasing the risk of pregnancy loss (38, 39).

Thyroid hormones are composed primarily of T3 and T4, with T3 being the more active form. In the placental extravillous trophoblasts (EVTs) during early pregnancy, T3 enhances the expression of MMP-2, fetal fibronectin, MMP-3, and integrin α5β1. These proteins are essential for placental growth and the ability of the EVTs to invade (40). Elevated T3 levels, particularly when accompanied by elevated TSH levels, may indicate hypothyroidism, which increases the risk of pregnancy loss (41). Elevated FT4 levels are typically associated with hyperthyroidism, and abnormally elevated pre-pregnancy FT4 levels may affect endocrine homeostasis and the process of embryo implantation. Previous studies have reported that hyperthyroid women exhibit elevated follicle-stimulating hormone, estradiol, and luteinizing hormone levels, which may increase the risk of early embryonic arrest and pregnancy loss (42). Furthermore, immune abnormalities linked with thyroid diseases, particularly thyroid autoimmunity (TAI), are connected to an elevated risk of RPL in women (43). TAI can induce immunological dysfunction and impede immune tolerance at both systemic and maternal-fetal interface levels (44). Additionally, thyroid autoantibodies, particularly TGAb, could be an expression of a more general maternal immune system abnormality leading to RPL (45). Research has shown that a positive TGAb and TPOAb expression is also associated with a higher incidence of hypothyroidism (46), with the presence of TPOAb being linked to RPL (47). Therefore, TAI and the thyroid function may have significant implications for the pathophysiology of RPL.

Overall, these findings emphasize the importance of thyroid functional status for pregnancy maintenance, especially in RPL women. Maintaining optimal maternal thyroid hormone levels is crucial in balancing the inflammatory response during early pregnancy by regulating the secretion of essential cytokines and angiogenic growth factors by the metaphase cells, and it helps promote normal placental development (48). Therefore, it is important for RPL women who are planning to become pregnant to undergo thyroid function test and receive appropriate management for any thyroid disorders. Further research is required to fully understand the correlation between pre-pregnancy thyroid-related hormones and RPL.

In this study, we found that RPL patients who had lower LY30 values and higher EPL values were at a greater risk of experiencing subsequent pregnancy loss. Rai et al. also reported a significant decrease in LY30 among women with RPL (21). LY30 is a measure of thrombolysis within 30 minutes of thrombosis and is used to evaluate fibrinolytic activity. Decreased LY30 indicates decreased fibrinolytic activity and increased clot stability, which may lead to a greater tendency for the blood to form a persistent thrombus (49). Any factor that affects blood circulation in the uterus may increase the risk of pregnancy loss since adequate blood supply is required for embryo implantation and early development (50). EPL is the estimated percentage of thrombolysis at the end of the TEG measurement, and an elevated EPL indicates strong fibrinolytic activity. Lower pre-pregnancy EPL values may indicate an underlying imbalance between the blood coagulation and fibrinolytic systems, which exists before pregnancy and may be exacerbated by physiological changes during pregnancy. This may lead to an increased risk of pregnancy loss, as normal regulatory mechanisms may be unable to adapt. The current findings suggest that TEG parameters to assess the pre-pregnancy coagulation state of RPL patients are important in improving the risk of subsequent pregnancy outcomes. Meanwhile, further studies are needed to investigate the mechanisms behind this association.

Risk prediction models have become increasingly common in the study of complex diseases. In this study, we utilized LASSO and stepwise regression analyses to select variables and construct a model with good performance using a minimal number of characteristics. Also, a dynamic nomogram was established to display the predictive model in an interactive and user-friendly way, and the external validation of the model’s predictive performance yielded good results. Our model is the first tool developed to predict the risk of subsequent pregnancy loss in patients with RPL based on thyroid function and TEG examination parameters. A web version of the model was also created for convenient utilization by clinicians and patients. At the same time, we constructed a risk stratification that categorizes patients into subgroups with high or low risk of pregnancy loss, which makes it easier and more intuitive to help clinicians make the appropriate treatment decisions.

Nevertheless, this study has some limitations. This retrospective study was inevitably subject to selection bias. Besides, the treatment information of these patients was not included and analyzed in this study, and it is unclear whether the patient’s previous treatment had an impact on pregnancy outcomes. Other studies found that lifestyle modification and supportive treatment are related to a live birth outcome (51, 52). Moreover, the relationship between the included variables and RPL is not well understood and remains to be further explored and elucidated in future work. Finally, the differences in RPL definitions also make the generalizability of this model somewhat limited (53). Since the RPL population in this study was included in accordance with the ESHRE RPL guidelines, patients matching this RPL definition may benefit more from this model. Future studies can continue to investigate the applicability of this prediction model under different RPL definitions to further validate our findings.

## 5 Conclusions

In the present study, we developed a nomogram to predict the risk of subsequent pregnancy loss in RPL patients using the pre-pregnancy thyroid function and TEG examination parameters, and external validation further confirmed that the model exhibits good performance. The web-based version of this model was user-friendly. This nomogram could help clinicians identify high-risk patients and make informed decisions regarding the management of subsequent pregnancies in RPL.

## Data availability statement

The raw data supporting the conclusions of this article will be made available by the authors, without undue reservation.

## Ethics statement

The studies involving humans were approved by Ethics Committee of Lanzhou University Second Hospital. The studies were conducted in accordance with the local legislation and institutional requirements. The participants provided their written informed consent to participate in this study.

## Author contributions

FM: Conceptualization, Writing – original draft. HH: Writing – original draft, Data curation, Formal analysis. CW: Data curation, Formal analysis, Writing – original draft. NH: Writing – original draft, Visualization. FW: Conceptualization, Writing – review & editing.
