# Corrigendum: A new prognostic model for recurrent pregnancy loss: assessment of thyroid and thromboelastograph parameters

**DOI:** 10.3389/fendo.2024.1447049

**Published:** 2024-06-20

**Authors:** Fangxiang Mu, Huyan Huo, Chen Wang, Ning Hu, Fang Wang

**Affiliations:** Department of Reproductive Medicine, Lanzhou University Second Hospital, Lanzhou, China

**Keywords:** recurrent pregnancy loss, thyroid function, thromboelastograph, nomogram, prediction model

**Error in Figure/Table**

In the published article, there was an error in [**Table 2** and **Table 3**] as published. [**Table 2** and **Table 3** are in the wrong order]. The corrected [**Table 2** and **Table 3**] and its caption appear below.

**Table 2:** Selecting predictive variables using LASSO and stepwise regression.

Variables	LASSO regression	Stepwise regression
Maternal age	True	
BMI	True	True
Previous pregnancy losses	True	True
T3	True	True
FT4	True	True
TSH	True	True
TG	True	
R	True	
K	True	
LY30	True	True
EPL		True
AIC value	611.7	605.1
Number of predictors	10	7

BMI, body mass index; T3, triiodothyronine; FT4, free thyroxine; TSH, thyroid stimulating hormone; TG, thyroglobulin; R, reaction time; K, kinetic time; LY30, lysis at 30 minutes; EPL, estimated percent lysis; AIC, Akaike information criterion.

**Table 3:** Multivariate logistic regression for the association between final predictors and subsequent pregnancy loss in RPL.

Variables	OR (95%CI)	*P* value
BMI	1.175 (1.110-1.258)	<0.001
Previous pregnancy losses		
Ref	–	
=3	1.583 (0.934-2.658)	0.084
≥4	1.873 (0.946-3.668)	0.068
T3	0.616 (0.333-1.086)	0.106
FT4	1.056 (0.981-1.139)	0.143
TSH	2.369 (1.891-3.002)	<0.001
LY30	0.216 (0.069-0.610)	0.046
EPL	2.938 (1.136-8.051)	0.025

Data are presented as odds ratio (OR) and 95% confidence interval (CI). BMI, body mass index; T3, triiodothyronine; FT4, free thyroxine; TSH, thyroid stimulating hormone; LY30, lysis at 30 minutes; EPL, estimated percent lysis.

The authors apologize for this error and state that this does not change the scientific conclusions of the article in any way. The original article has been updated.

