# Supplementary material for: A new prognostic model for recurrent pregnancy loss: assessment of thyroid and thromboelastograph parameters
Source: Front Endocrinol (Lausanne). 2024 May 30;15:1415786. doi: 10.3389/fendo.2024.1415786 (PMC11177760; doi:10.3389/fendo.2024.1415786)
Supplement: Supplementary file 1 [file DataSheet_1.docx]

**Supplementary materials**

**Figure S1.** Patients’ selection of training cohort.

**
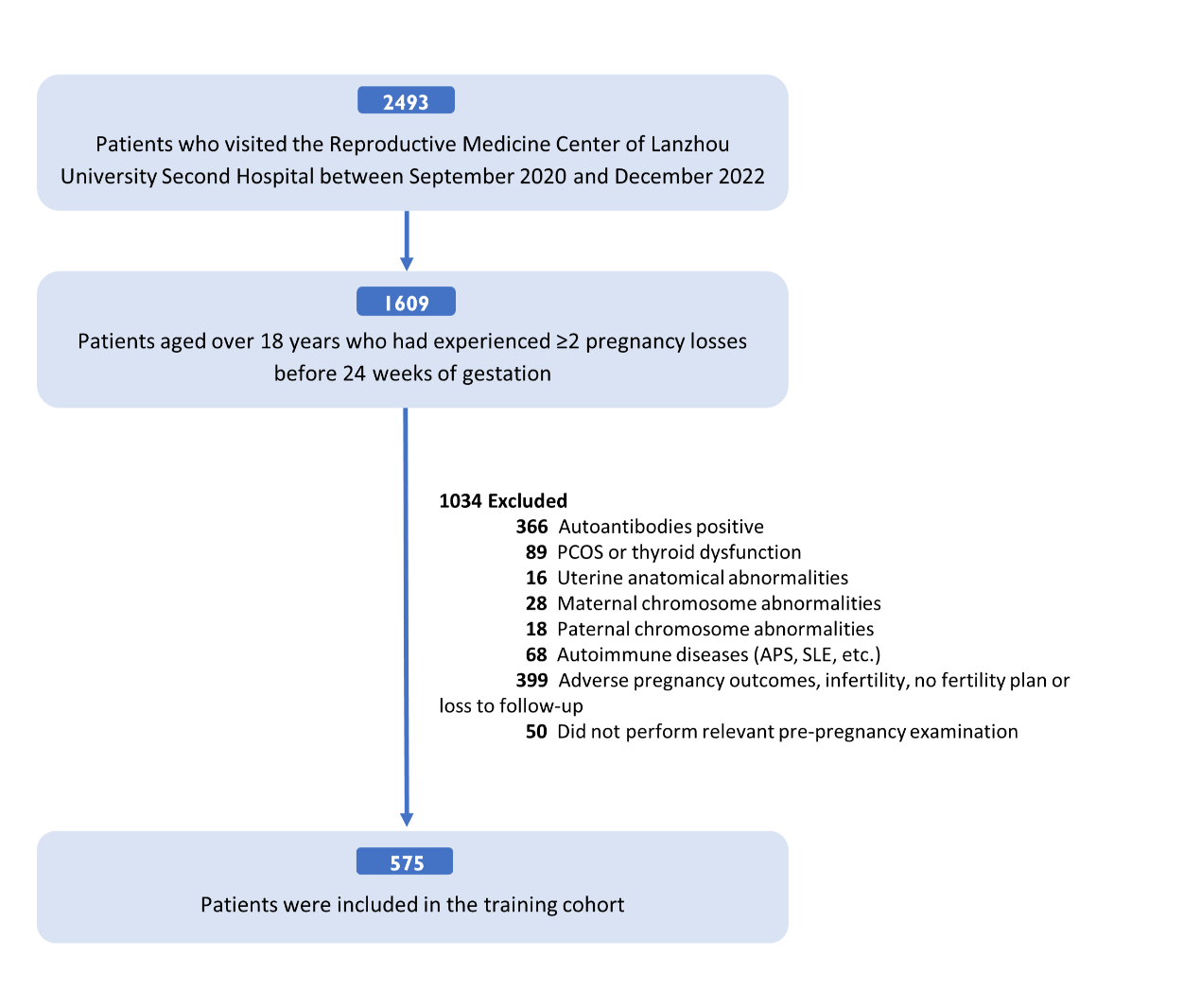
**

**Figure S2.** Patients’ selection of validation cohort.

**
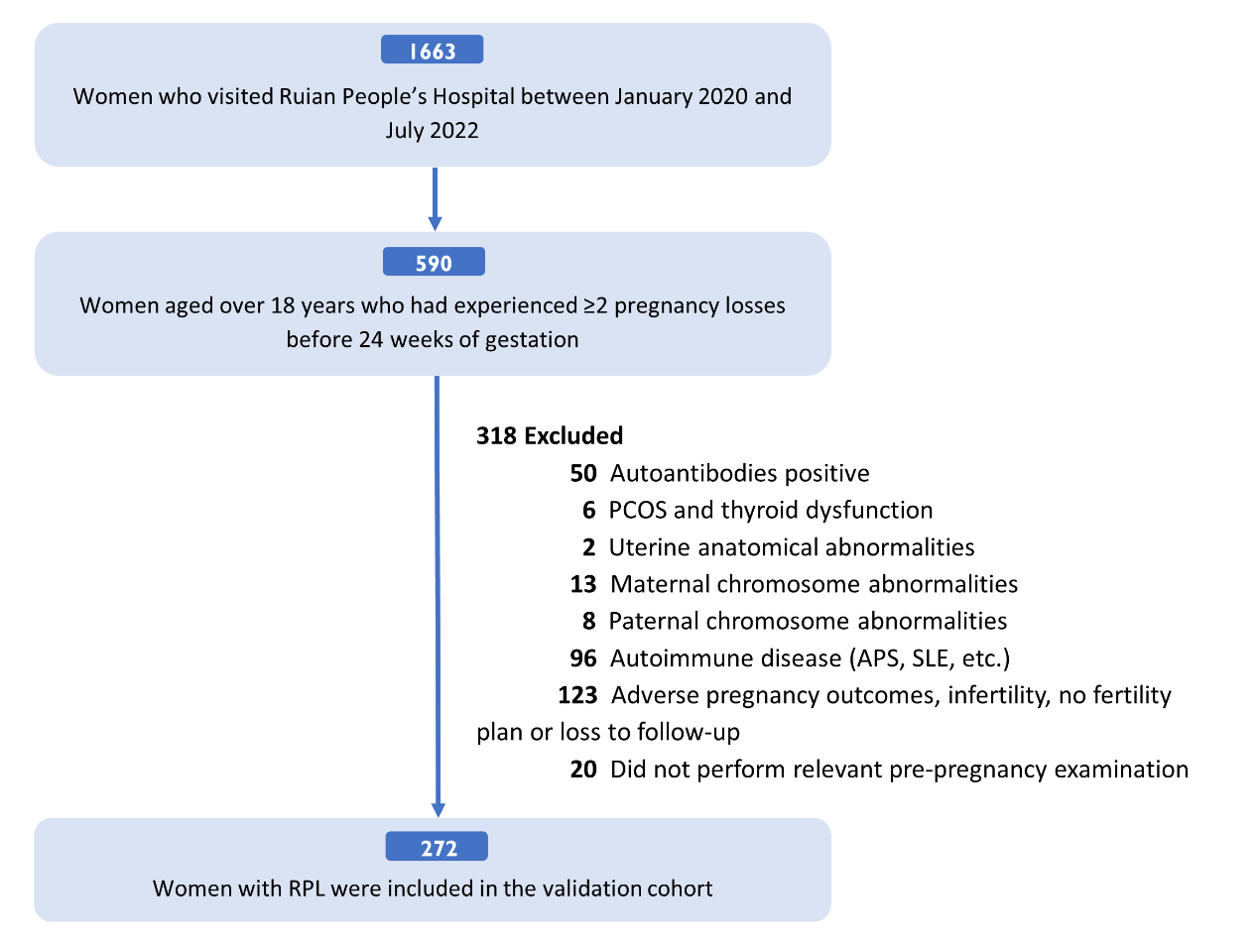
**

**Figure S3.** Calibration and clinical usefulness of the nomogram in the validation cohort.

**
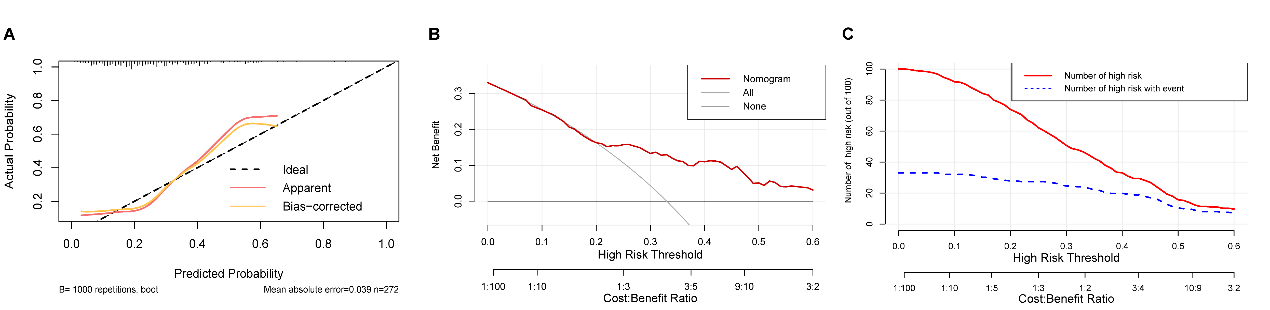
**

**A.** Calibration curve. **B.** Clinical decision curve. **C.** Clinical impact curve.

**Table S1.** Comparison of baseline and clinical characteristics between training cohort and validation cohort.

| **Variables** | **Training cohort (n=575)** | **Validation cohort (n=272)** | ***P* value** |
| --- | --- | --- | --- |
| Maternal age, years | 30.69±4.18 | 31.23±4.82 | 0.114 |
| BMI, kg/m^2^ | 22.33±2.99 | 22.48±4.02 | 0.583 |
| Previous pregnancy losses |  |  | <0.001 |
| 2 | 427 (74.3) | 159 (58.5) |  |
| 3 | 94 (16.3) | 70 (25.7) |  |
| ≥4 | 54 (9.4) | 43 (15.8) |  |
| T3, nmol/L | 1.81±0.36 | 1.70±0.37 | <0.001 |
| T4, nmol/L | 111.91±23.76 | 116.87±27.28 | 0.007 |
| FT3, pmol/L | 5.20±0.66 | 4.60±0.67 | <0.001 |
| FT4, pmol/L | 16.31±2.63 | 14.64±2.45 | <0.001 |
| TSH, μIU/mL | 2.05±0.97 | 1.90±1.90 | 0.224 |
| TG, ng/mL | 12.32±17.40 | 16.02±11.11 | 0.001 |
| TGAb (+) | 64 (11.1) | 29 (10.7) | 0.839 |
| TPOAb (+) | 66 (11.5) | 31 (11.4) | 0.972 |
| R, min | 5.83±2.52 | 5.55±2.82 | 0.161 |
| K, min | 2.88±7.48 | 1.47±0.79 | <0.001 |
| Angle, deg | 63.88±10.89 | 71.67±5.96 | <0.001 |
| MA, mm | 64.56±5.48 | 66.34±4.64 | <0.001 |
| LY30 | 0.41±0.97 | 1.85±3.28 | <0.001 |
| EPL, % | 0.43±1.02 | 5.88±2.63 | <0.001 |
| CI, % | 0.63±1.94 | 1.62±2.79 | <0.001 |

**Note:** Data are presented as mean ± standard deviation or frequencies with percentages. BMI, body mass index; T3, triiodothyronine; T4, thyroxine; FT3 free triiodothyronine; FT4, free thyroxine; TSH, thyroid stimulating hormone, TG, thyroglobulin; TGAb, antithyroglobulin antibody; TPOAb, thyroid peroxidase antibody; R, reaction time; K, kinetic time; MA, maximum amplitude; LY30, lysis at 30 minutes; EPL, estimated percent lysis; CI, coagulation index.
